# A Three-Marker FISH Panel Detects More Genetic Aberrations of *AR*, *PTEN* and *TMPRSS2/ERG* in Castration-Resistant or Metastatic Prostate Cancers than in Primary Prostate Tumors

**DOI:** 10.1371/journal.pone.0074671

**Published:** 2013-09-30

**Authors:** Xiaoyu Qu, Grace Randhawa, Cynthia Friedman, Brenda F. Kurland, Lena Glaskova, Ilsa Coleman, Elahe Mostaghel, Celestia S. Higano, Christopher Porter, Robert Vessella, Peter S. Nelson, Min Fang

**Affiliations:** 1 Fred Hutchinson Cancer Research Center, Seattle, Washington, United States of America; 2 Seattle Cancer Care Alliance, Seattle, Washington, United States of America; 3 University of Washington, Seattle, Washington, United States of America; 4 Virginia Mason Medical Center, Seattle, Washington, United States of America; 5 Puget Sound VA Health Care System, Seattle, Washington, United States of America; The University of Texas M.D Anderson Cancer Center, United States of America

## Abstract

*TMPRSS2*/*ERG* rearrangement, *PTEN* gene deletion, and androgen receptor (*AR*) gene amplification have been observed in various stages of human prostate cancer. We hypothesized that using these markers as a combined panel would allow better differentiation between low-risk and high-risk prostate cancer. We analyzed 110 primary prostate cancer samples, 70 metastatic tumor samples from 11 patients, and 27 xenograft tissues derived from 22 advanced prostate cancer patients using fluorescence in situ hybridization (FISH) analysis with probes targeting the *TMPRSS2*/*ERG*, *PTEN*, and *AR* gene loci. Heterogeneity of the aberrations detected was evaluated. Genetic patterns were also correlated with transcript levels. Among samples with complete data available, the three-marker FISH panel detected chromosomal abnormalities in 53% of primary prostate cancers and 87% of metastatic (Met) or castration-resistant (CRPC) tumors. The number of markers with abnormal FISH result had a different distribution between the two groups (*P*<0.001). At the patient level, Met/CRPC tumors are 4.5 times more likely to show abnormalities than primary cancer patients (*P*<0.05). Heterogeneity among Met/CRPC tumors is mostly inter-patient. Intra-patient heterogeneity is primarily due to differences between the primary prostate tumor and the metastases while multiple metastatic sites show consistent abnormalities. Intra-tumor variability is most prominent with the *AR* copy number in primary tumors. *AR* copy number correlated well with the *AR* mRNA expression (rho = 0.52, *P*<0.001). Especially among *TMPRSS2:ERG* fusion-positive CRPC tumors, *AR* mRNA and *ERG* mRNA levels are strongly correlated (rho = 0.64, *P*<0.001). Overall, the three-marker FISH panel may represent a useful tool for risk stratification of prostate cancer patients.

## Introduction

The discovery of recurrent *ETS* gene rearrangements in prostate cancers has led to studies evaluating the functional role of *ETS* genes in the pathogenesis of this disease and as diagnostic and prognostic biomarkers. The most common type of *ETS* rearrangement, the fusion of androgen-regulated *TMPRSS2* with the oncogenic *ERG* is detected in approximately half of prostate tumors but none of benign glands [1]. However, studies assessing the prognostic significance of *TMPRSS2*:*ERG* fusion have yielded inconsistent results [2]–[5]. Additional genetic factors are likely to work in concert with the fusion during cancer progression. Recent studies have shown that genetic aberrations are not only common in prostate cancer but also interact with each other through related pathways, thereby contributing to the progression to invasive diseases. *TMPRSS2* is regulated by androgens, and the androgen receptor (AR) is often amplified in patients treated with androgen deprivation therapy [6], [7]. *PTEN* deletion, another common aberration in prostate cancer, was correlated with the expression of downstream p-Akt and associated with cancer-specific mortality [8], [9]. *ETS* gene rearrangements were shown to cooperate with *PTEN* deletion and impact prostate cancer prognosis [10], [11]. Crosstalk between PI3K and AR signaling pathways was recently suggested as a mechanism for the development of castration resistant prostate cancer (CRPC) [12], [13]. *PTEN* deletion was shown to suppress androgen-responsive gene expression by modulating *AR* transcription factor activity. Also, *PTEN* and *AR* expression has been shown to inversely correlate in prostate cancer [14].

A critical clinical question concerns identifying characteristics of newly diagnosed prostate cancers that will distinguish aggressive from indolent behavior. The molecular heterogeneity of prostate cancers suggests that individual biomarkers may not be sufficient, and that multiple genetic markers may better associate with outcome. In the present study, we used a three-marker fluorescence in situ hybridization (FISH) panel to detect *TMPRSS2* and/or *ERG* rearrangements, *AR* gene amplification, and *PTEN* deletion in both primary and CRPC prostate cancer samples and compared the prevalence, concurrence, and interaction of these three markers. With the reference of mRNA expression data generated from matching tumor samples from the same patient, we also demonstrated how FISH findings correlated with changes in gene expression. Intra- and inter-patient tumor heterogeneity was also analyzed.

## Materials and Methods

### Sample Acquisition

#### Ethics Statement

The study was approved by the Institutional Review Boards (IRB) of the Fred Hutchinson Cancer Research Center and the University of Washington Medical Center. IRB waived the need for written consent for this study because only de-identified materials were used, which were from the University of Washington Urology tissue bank.

#### Patient samples

De-identified archived untreated primary prostate cancer samples (n = 110) were obtained from the University of Washington (UW) and Virginia Mason Hospital in Seattle. A total of 83 primary tumors generated analyzable data for at least one FISH marker in the panel, including 69 patients with *TMPRSS2*/*ERG* FISH data, 65 patients with *AR* FISH data and 42 patients with *PTEN* FISH data. Metastatic tumor samples (n = 70) were collected at UW from autopsies performed within 2 to 4 hours of death of 11 CRPC patients under the rapid autopsy program [15]. Tumors were obtained from various organ sites, frozen immediately and stored at −80°C. All tissues were sectioned for H&E staining and, for verification of histology, reviewed by a pathologist. FISH analysis was focused on cancer areas. A total of 67 tumors yielded analyzable data for at least one FISH marker in the panel, including 56 tumors from 10 patients with *TMPRSS2*/*ERG* FISH data, 65 tumors from 11 patients with *AR* FISH data, and 62 tumors from 11 patients with *PTEN* FISH data.

#### Prostate cancer xenografts

Prostate cancer xenografts (LuCaP lines) were originally isolated from various organs of advanced patients [16]. FISH analyses were successful on 27 LuCaP lines, representing 22 patients, one of which was also among the metastatic patients described above. These included 27 tumors from 22 patients with *TMPRSS2*/*ERG* FISH data, 26 tumors from 21 patients with *AR* FISH data, and 25 tumors from 21 patients with *PTEN* FISH data. Together, combining metastatic patient tumors and xenografts derived from advanced-stage prostate cancer patients, the current study evaluated a total of 94 tumors from 32 patients.

### Fluorescent In Situ Hybridization (FISH)

*TMPRSS2*/*ERG* rearrangement was assessed using our novel 4-color FISH assay as described separately [17]. “*TMPRSS2:ERG*” refers to the presence of fusion of the two genes. “*TMPRSS2*/*ERG* rearrangement” refers to various subtypes of rearrangement of either or both genes as specified in the Results section. FISH analysis of *AR* gene amplification was performed using the SpectrumOrange AR (Xq12) probe combined with the SpectrumGreen labeled ChrX centromere (Xp11.1-q11.1) CEP X probe as the control (Abbott Molecular, IL). *PTEN* gene deletion was examined using the *PTEN*/CEP10 dual-color FISH Probe set (Abbott Molecular, IL), including the SpectrumOrange labeled PTEN (10q23) probe and the SpectrumGreen labeled Chr10 centromere (10p11.1–10q11.1) CEP 10 probe.

For each sample, a range of 25 to 50 intact and non-overlapping interphase nuclei were enumerated manually using a 100×oil immersion lens on a Zeiss Z1 microscope (Carl Zeiss Canada Ltd, Canada). *AR* gain and *PTEN* deletion were assessed by counting the number of gene signal and the corresponding centromere signal per nucleus. *AR* gain was defined as an average copy number of *AR* per nuclei equal or higher than 2. True *AR* gene amplification was defined as the ratio of the total number of *AR* signals divided by the total number of the X-chromosome centromere equal or greater than 2. Samples with *PTEN* heterozygous deletion had a ratio of the total number of *PTEN* signals divided by the total number of CEP10 signals equal or below 0.75. A *PTEN*/CEP10 ratio equal or below 0.2 is considered homozygous *PTEN* deletion. For patient-level analyses of CRPC patients with multiple tumors, expression by a given marker was considered abnormal if the aberration was seen in at least one tumor.

### Expression Array

Agilent 44 K whole human genome expression oligonucleotide microarrays (Agilent Technologies, Inc., Santa Clara, CA) were used to profile prostate cancer xenografts and human castration-resistant soft tissue metastases of prostate. Freshly frozen xenografts were processed to extract total RNA which was amplified one round; patient samples were laser-capture microdissected and amplified two rounds as described previously [18]. Probe labeling and hybridization was performed following the Agilent suggested protocols and fluorescent array images were collected using the Agilent DNA microarray scanner G2565BA. Agilent Feature Extraction software was used to grid, extract, and normalize data. Expression ratios were log_2_ scaled and mean-centered across each gene.

### Statistical Analysis

To complement the comparisons of archived primary tumor with a separate cohort of patients with metastatic disease, we examined within-patient heterogeneity of *AR* and *PTEN* for patients with metastatic disease, hypothesizing that prostate tumors could differ from contemporaneous metastatic lesions. Linear mixed models with random patient effects were fitted to non-prostate tumors, and a 95% confidence interval calculated for subject-specific [19] predictions of average expression. If a subject’s prostate tumor copy number status fell outside the confidence interval, it would be interpreted as evidence of potential differences between the copy number status of primary and metastatic lesions. A linear mixed effects model and the %ICC9 SAS macro was used to calculate intraclass correlation coefficients and their confidence intervals [20]. Logistic regression and generalized estimating equations (GEE) were used to compare rates of abnormality for primary and metastatic samples, controlling for tissue source (rapid autopsy vs xenograft) and within-patient correlation for tumor-level analysis. Heterogeneity of intratumoral variance for different tumor sites was also explored using linear mixed models. Additional statistical inference included Spearman correlation coefficients, and the Wilcoxon rank sum test to compare distributions of the number of markers with abnormal expression. *P*-values were two-sided; statistical analyses were conducted using SAS/STAT software, version 9.3 (SAS Institute, Inc., Cary, NC).

## Results

### The Prevalence of Genetic Aberrations Detected by the Three-marker FISH Panel in Localized Primary and Metastatic or Castration Resistant (Met/CRPC) Patients

**Figure 1:**
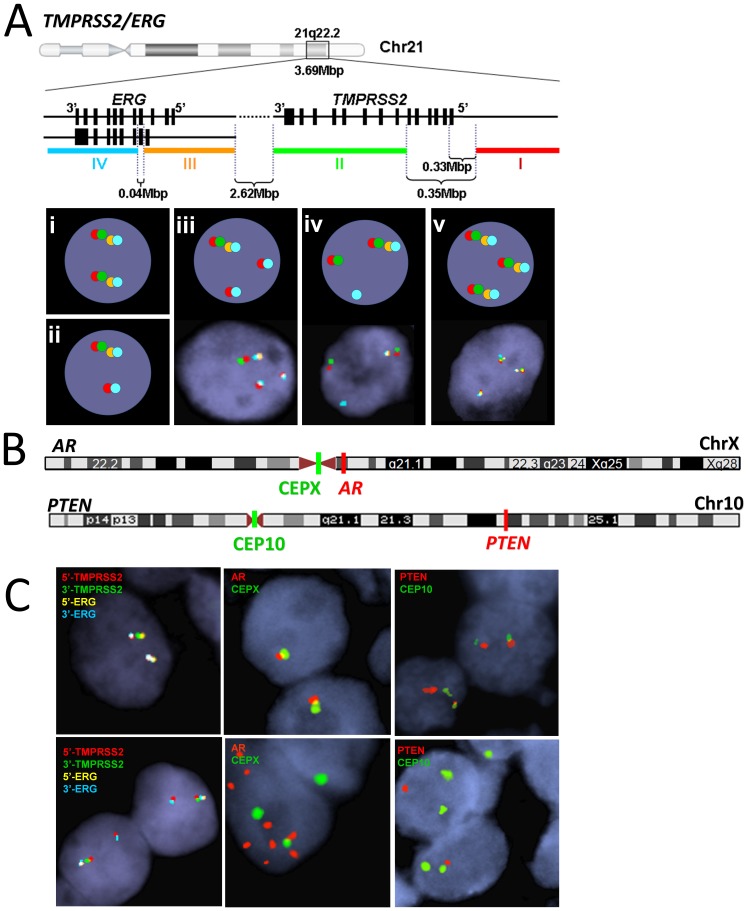
The three-marker FISH panel including *TMPRSS2*/*ERG* rearrangements, *AR* gene amplification, and *PTEN* gene deletion. (A) Illustration of the 4-color FISH technique for the detection of rearrangements of *TMPRSS2* and/or *ERG*. FISH probes target 5′-*TMPRSS2* (red, probe I), 3′-*TMPRSS2* (green, probe II), 5′-*ERG* (gold, probe III), and 3′-*ERG* (blue, probe IV) simultaneously, detecting various signal patterns including normal (i), single fusion(ii), dual/complex fusion(iii), alternative rearrangement without fusion (iv), and copy number increase(CNI) without rearrangements. Captured FISH images of (i) and (ii) are shown in the left panel of 1C; images of (iii) – (v) are shown below the corresponding illustration. (B) FISH probes used to detect *AR* gene amplification and *PTEN* gene deletion *AR* gene amplification was analyzed using probes targeting *AR* (orange) and the X-chromosome centromere (green, CEPX). *PTEN* gene deletion was detected using probes targeting *PTEN* (orange) and the chromosome 10 centromere (green, CEP10). (C) Representative interphase FISH images. Top left, normal *TMPRSS2* and *ERG* signal pattern demonstrating two sets of the four probes per nucleus; Bottom left, *TMPRSS2*: *ERG* fusion shown as juxtaposed red and blue signals concurrent with missing or separation of the interstitial green and gold signals; Top middle, normal *AR* signal pattern demonstrating one orange *AR* and one green X signal per nucleus; Bottom middle, *AR* gene amplification presenting more than twice the number of *AR* signals than the CEPX signals; Top right, normal *PTEN* signal pattern demonstrating 2 orange *PTEN* and 2 green CEP10 signals per nucleus; Bottom right, *PTEN* deletion showing none or 1 copy of *PTEN* signals per nucleus.

**Figure 2:**
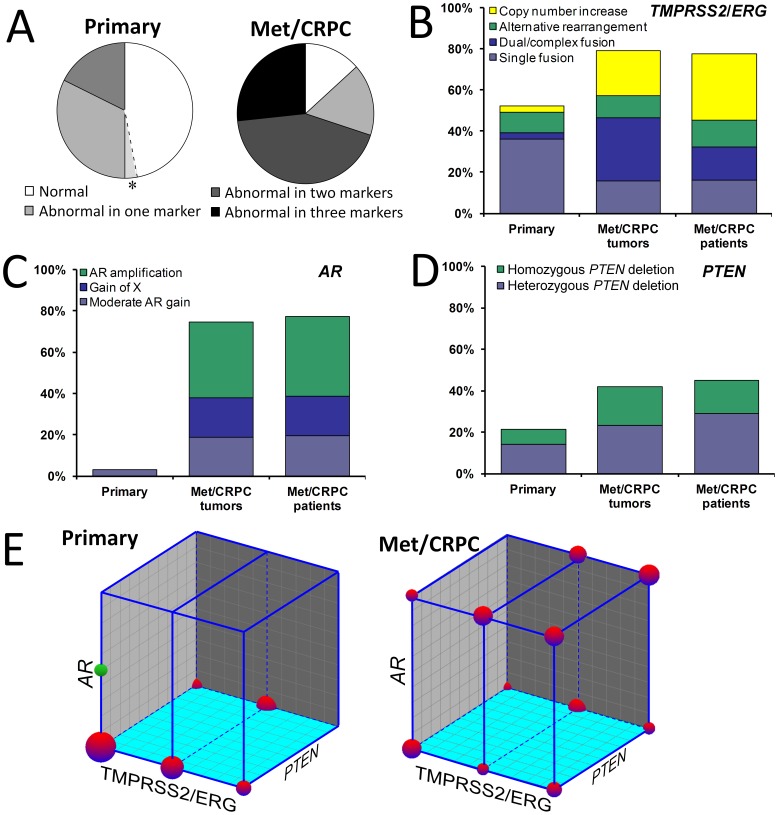
The prevalence of genetic aberrations detected by the panel. A patient with multiple tumors was considered abnormal by a given marker if the aberration was seen in at least one tumor. (A) Pie charts demonstrate the percentage of individuals with no (white), one (light grey), two (dark grey), and three (black) abnormalities detected by the panel among the primary prostate cancer (n = 34) and the metastatic or castration resistant prostate cancer (Met/CRPC) cohort (n = 30), respectively. Among primary patients, an asterisk was used to highlight moderate AR gain (average *AR* per nucleus > = 1.5 but <2). (B-D) Prevalence of each subtype of abnormalities detected by individual FISH marker among the primary patients (one tumor per patient, n = 34), Met/CRPC tumors (n = 81), and Met/CRPC patients/xenografts (n = 30), respectively. *TMPRSS2*/*ERG* abnormalities are categorized as single fusion (light blue), dual/complex fusion (dark blue), alternative rearrangements (green), and copy number increase (CNI) of the normal gene alleles (yellow). *AR* FISH detected moderate *AR* gain (light blue), gain of X (dark blue) and *AR* gene amplification (green). *PTEN* FISH abnormalities includes heterozygous (light blue) and homozygous (green) *PTEN* deletions. (E) The co-occurrence of abnormalities in the three markers shown as 3D sphere plots for the primary cancer cohort (left) and the Met/CRPC cohort (right). *TMPRSS2*/*ERG*, *PTEN*, and *AR* results are presented on X, Y, and Z axes, respectively. The value presented on each axis ranges from 0 to 1. “0” denotes normal for a given marker. For *TMPRSS2*/*ERG*, “0.5” indicates rearrangements, including fusion and alternative rearrangements; “1” means CNI of the normal alleles without any rearrangement. For *PTEN* FISH, both heterozygous and homozygous deletions are presented as “1”. For *AR* FISH, “1” indicates *AR* copy number gain (> = 2.0). Patients with the same combination of abnormalities are clustered into a sphere, the volume of which is proportional to the percentage of patients in the respective cohort. Only patients with available data from all three markers are included. The green sphere in the primary patient plot denotes moderate *AR* gain (>1.5 but <2.0).

The three-marker FISH panel (Figure 1) used in our study detected frequent genetic aberrations in prostate cancer, and these were significantly more common in Met/CRPC tumors than in untreated primary tumors (Figure 2A).

Of the 34 primary tumors in which all 3 markers could be assessed, 16 (47%) exhibited no aberrations involving *AR*, *PTEN* or *TMPRSS2/ERG*; 11 (32%) were abnormal by one marker only. Six patients’ tumors (18%) were detected abnormal by two markers, including 3 with *TMPRSS2*:*ERG* fusion and homozygous *PTEN* deletion, 2 with *TMPRSS2*:*ERG* fusion and heterozygous *PTEN* deletion, and 1 with non-fusion alternative rearrangement along with heterozygous *PTEN* deletion. None of the patients were abnormal by all three markers because there was no detectable *AR* abnormality when the cutoff for *AR* gain was set to > = 2.0 *AR* per nucleus, an arbitrarily determined stringent cutoff. Two patients would be classified as mild *AR* gain if using *AR* copy number per nuclei *>* = 1.5 as the cutoff value, established as mean+3SD based on enumeration results on normal prostate epithelial cells from 18 different samples.

Of the 30 Met/CRPC patients/xenografts with FISH results from all three markers, 4 (13%) had no abnormal marker values. Five (17%) were shown as abnormal by one marker only; 13 (43%) were detected as abnormal by two markers, including 8 (27%) shown as abnormal by *TMPRSS2*/*ERG* and *AR* FISH and 5 (17%) by *TMPRSS2*/*ERG* and *PTEN*. Eight patients (27%) were abnormal by all three tests.

We further evaluated subtypes of genetic aberrations detected by each marker in the Met/CRPC cohort (Figure 2 B–D). Rearrangements of *TMPRSS2* and/or *ERG* were detected in 14 patients (47%), including 5 (17%) with the typical single *TMPRSS2*:*ERG* fusion, 5 (17%) with dual or complex *TMPRSS2*:*ERG* fusion, and 4 (13%) with alternative rearrangements without fusion. Copy number increase (CNI) of chromosome 21 was observed in 10 patients (33%) using the *TMPRSS2*/*ERG* FISH probes. *AR* gain in one or more lesions was observed in 18 patients (60%), including 6 (20%) that resulted from gain of the X-chromosome and 12 (40%) with true *AR* gene amplification (*AR*/X > = 2). Deletion of *PTEN* was detected in 15 patients (50%), including 5 with homozygous deletion.

A Wilcoxon rank sum test suggested that the Met/CRPC cohort (n = 30) generally had more alterations detected by FISH than the cohort of primary cancers (N = 34) (W = 1287, *P*<0.001). *AR* gain, including moderate gain (W = 1334, *P*<0.001), and the combination of *TMPRSS2/ERG* and *PTEN* alterations (W = 1181, *P* = 0.005) were also significantly more common in the Met/CRPC tumors.

The investigation of individual markers reflected unique trends of changes of each genetic abnormality during the progression of prostate cancer. About 80% of Met/CRPC samples were identified by *TMPRSS2*/*ERG* FISH as abnormal, compared to 48% in primary samples, and the difference was statistically significant (Table 1; Figure 2B) (*P* = 0.03). This difference appeared to be due to the CNI aberration rather than the *TMPRSS2:ERG* fusion itself; the percentage of patients with fusion or alternative rearrangement remains similar, but the percentage of patients with dual fusion as opposed to single fusion is clearly greater in the Met/CRPC category than in the primary tumor group (Figure 2B). Examining individual tumors (adjusting for within-person correlation and xenograft status), the odds of a Met/CRPC tumor exhibiting an abnormality was 4.5 times greater than odds for a primary tumor (*P* = 0.05). While nearly all primary cancer patients showed normal *AR* status, over 70% of Met/CRPC patients demonstrated various degrees of *AR* gene copy number gain (Table 1; Figure 2C). PTEN FISH showed increased heterozygous *PTEN* deletion and homozygous *PTEN* deletion in Met/CRPC compared with primary patients (Table 1; Figure 2D) (*P = *0.07 at the tumor level, *P = *0.003 at the patient level). Of note, for patient-level assessments there was a hierarchy, so if one lesion was heterozygous and the other homozygous, the patient level was considered homozygous.

**Table 1:** Prevalence of abnormalities detected by each FISH marker among the primary and the metastatic or castration-resistant prostate cancer (Met/CRPC) patients.

	Primary		Met/CRPC		Met/CRPC		*P*value	
	# ofpatients	%	# oftumors	%	# ofpatients	%	Tumors^6^	Patients^7^
***TMPRSS2/ERG***	69		82		31		0.05	0.03
Single fusion	25	36%	13	16%	5	16%		
Dual/complex fusion	2	3%	25	30%	5	16%		
Alternative rearrangementwithout fusion	4	6%	9	11%	4	13%		
Copy number increase	2	3%	18	22%	10	32%		
Normal	36	52%	17	21%	7	23%		
***AR***	65		90		31		<0.001	<0.001
Moderate A*R* gain^1^	2	3%	17	19%	6	19%		
Gain of X^2^	0	0%	17	19%	6	19%		
*AR* amplification^3^	0	0%	33	37%	12	39%		
Normal	63	97%	23	26%	7	23%		
***PTEN***	42		86		31		0.07	0.003
Heterozygous*PTEN* deletion^4^	6	14%	20	23%	9	29%		
Homozygous*PTEN* deletion^5^	3	7%	16	19%	5	16%		
Normal	33	79%	50	58%	17	55%		

1 Average *AR* per nucleus ≥1.5 but <2.

2 Average *AR* per nucleus ≥2 but average AR/X ratio<2.

3 Average *AR/*X ratio≥2.

4 Average *PTEN*/CEP10 ratio≤0.75 but >0.2.

5 Average *PTEN*/CEP10 ratio≤0.2.

6 Wald tests of abnormal vs. normal for primary vs. CRPC, generalized estimating equations (GEE) with independence autocorrelation, adjusting for rapid autopsy vs xenograft sample for CRPC. Likelihood ratio test for AR (without adjustment for autocorrelation), since no primary samples had abnormal AR.

7 Wald tests of abnormal vs. normal for primary vs. CRPC, logistic regression adjusting for rapid autopsy vs xenograft sample for CRPC. Likelihood ratio test for AR, since no primary samples had abnormal AR.

Data of the entire panel across different individuals showed that prostate cancer patients with *PTEN* deletion also tended to exhibit abnormal results in *TMPRSS2*/*ERG* FISH (Figure 2E). Among the 34 primary cancer patients with data available from all three markers, 6 of the 8 individuals with *PTEN* deletion (75%) also showed an abnormal *TMPRSS2*/*ERG* FISH result (Figure 2E). Among the 30 Met/CRPC patients with data available from with either both or all three markers, 13 of the 14 individuals with *PTEN* deletion (93%) also showed abnormalities in *TMPRSS2*/*ERG* FISH analysis. In Met/CRPC patients, abnormal *TMPRSS2/ERG* FISH results were also more prevalent among patients demonstrating gain of *AR*, and vice versa (Figure 2E). Sixteen out of 17 patients (94%) with *AR* gain showed *TMPRSS2*/*ERG* abnormalities. Sixteen of 24 patients (67%) with *TMPRSS2*/*ERG* abnormalities also demonstrated *AR* gain. Detailed FISH results on all metastatic samples from each CRPC patient are summarized in Table 2. Results of xenograft samples are listed in Table 3.

**Table 2:** FISH data of individual castration resistant metastatic patient tumors.

Patient	Tissue	*TMPRSS2/ERG*		Average *AR*per nucleus	*AR*/*X*	*PTEN*/*CEP10*
8	Liver	Normal		5.50	3.27	1.19
8	LN1	Normal		1.12	1.00	1.04
8	LN2	Normal		4.94	2.74	1.20
8	LN3	Normal		5.70	3.35	1.16
8	Lung	Normal		4.38	2.74	1.12
8	Prostate	Normal		2.52	1.42	1.00
1	Liver	Normal		NA	NA	0.50
1	LN1	Normal		1.00	1.00	0.52
1	LN2	Normal		1.03	1.00	0.49
1	Prostate	Copy number increase		1.00	1.00	0.50
4	Liver	Single fusion		1.19	1.00	0.00
4	LN1	Single fusion		1.05	0.98	0.00
4	Lung1	Single fusion		1.00	1.00	0.00
4	Lung2	NA		1.11	1.00	NA
4	Spleen	Single fusion		1.04	1.00	0.00
4	Prostate	NA		1.08	1.00	NA
5	LN1	Dual/complex fusion		20.37	7.10	0.04
5	LN2	Dual/complex fusion		20.76	6.18	0.05
5	LN3	Dual/complex fusion		37.48	10.18	0.00
5	LN4	Dual/complex fusion		18.16	6.78	0.08
5	LN5	Dual/complex fusion		14.48	6.58	0.10
5	Prostate	Single fusion		102.64	54.60	0.03
2	LN1	Dual/complex fusion		1.10	1.00	1.00
2	LN2	Dual/complex fusion		1.66	0.99	0.80
2	LN3	Dual/complex fusion		1.92	1.02	0.97
2	Lung1	Dual/complex fusion		1.22	1.00	0.96
2	Lung2	Dual/complex fusion		2.00	1.00	0.98
2	Prostate	Dual/complex fusion		1.75	1.00	0.95
9	Adrenal1	Dual/complex fusion		1.62	1.09	1.02
9	Adrenal2	Dual/complex fusion		1.62	1.09	1.02
9	Liver	Dual/complex fusion		1.50	0.99	0.96
9	LN1	Dual/complex fusion		1.86	1.06	0.96
9	LN2	Dual/complex fusion		1.50	0.96	1.00
9	LN3	Dual/complex fusion		1.28	0.98	1.02
9	LN4	Dual/complex fusion		1.26	1.02	0.90
9	Lung1	Dual/complex fusion		NA	NA	0.94
9	Lung2	Dual/complex fusion		1.48	1.04	1.04
9	Spleen	Dual/complex fusion		1.64	1.01	0.86
9	Prostate	Dual/complex fusion		14.22	6.35	0.95
7	LN1		Alternative rearrangement	8.20	3.20	0.73
7	LN2		Alternative rearrangement	16.48	9.81	0.80
7	LN3		Alternative rearrangement	17.32	8.33	0.82
7	LN4		Alternative rearrangement	37.64	12.38	0.50
7	Prostate		Alternative rearrangement	10.20	5.31	0.76
11	LN1		Copy number increase	6.88	3.91	0.03
11	LN2		NA	7.38	4.15	0.05
11	LN3		Copy number increase	7.44	4.33	0.06
11	Lung		NA	5.56	3.39	0.01
11	Prostate		Alternative rearrangement	1.00	1.00	0.94
11	Prostate		Alternative rearrangement	1.10	1.04	1.02
3	Liver		Copy number increase	2.58	1.16	0.94
3	LN1		Copy number increase	2.80	1.32	0.76
3	LN2		Copy number increase	2.88	1.29	0.82
3	Lung		Copy number increase	2.74	1.28	1.00
3	Prostate 1		Normal	4.04	3.61	0.64
3	Prostate 2		Normal	1.32	1.06	NA
6	LN1		Copy number increase	2.56	1.00	NA
6	LN2		Copy number increase	2.44	1.00	0.57
6	LN3		Copy number increase	2.55	1.00	NA
6	Peritoneal		Copy number increase	2.21	0.99	0.45
10	Liver		NA	2.88	1.73	0.51
10	LN1		NA	5.62	3.39	0.44
10	LN2		NA	5.35	3.54	0.51
10	LN3		NA	6.36	3.46	0.50
10	LN4		NA	9.78	5.62	0.50
10	Lung		NA	6.18	3.19	0.47
10	Prostate		NA	9.12	5.36	0.68

Only samples successfully hybridized with at least one marker were presented in the table, including 56 tumors with *TMPRSS2*/*ERG* FISH, 65 tumors with *AR* FISH, and 62 tumors with *PTEN* FISH results.

**Table 3:** FISH data of individual xenograft tumors.

Xenografts	Tissue	*TMPRSS2/ERG*	Average *AR*per nucleus	*AR*/*X*	*PTEN*/*CEP10*
LuCaP81	LN	Normal	1.00	1.00	0.91
LuCaP78	Peritoneal	Normal	1.04	1.00	1.00
LuCaP136	Acites fluid(cells)	Normal	1.04	1.00	0.00
LuCaP153†	NA	Normal	1.50	1.00	1.63
LuCaP147	Liver	Normal	1.96	1.96	1.00
LuCaP49	Omental fat met	Single fusion	1.10	0.97	0.54
LuCaP86.2	Bladder	Single fusion	1.97	1.00	0.93
LuCaP23.12	Liver	Single fusion	2.20	1.04	0.89
LuCaP23.1CR	LuCaP23.1	Single fusion	2.28	1.00	NA
LuCaP23.1	LN	Single fusion	2.48	1.00	0.95
LuCaP35	LN	Single fusion	6.44	2.98	0.91
LuCaP35CR	LuCaP35	Single fusion	34.76	12.78	0.96
LuCaP145.1*	Liver	Single fusion	1.60	1.00	1.15
LuCaP145.2*	LN	Dual/complex fusion	1.79	0.99	0.87
LuCaP93	Prostate	Dual/complex fusion	1.50	0.99	0.00
LuCaP92	Peritoneal	Dual/complex fusion	2.00	1.00	0.90
LuCaP58	LN	Alternative rearrangement	1.52	1.00	0.37
LuCaP96**	Prostate	Alternative rearrangement	1.52	1.03	0.60
LuCaP96CR	LuCaP96	Alternative rearrangement	5.72	3.33	0.79
LuCaP73	Prostate	Copy number increase	1.48	1.00	1.03
LuCaP115	LN	Copy number increase	1.72	1.08	1.02
LuCaP70	Liver	Copy number increase	2.08	1.00	1.00
LuCaP141	Prostate	Copy number increase	2.64	1.00	0.98
LuCaP146	NA	Copy number increase	6.80	3.90	1.16
LuCaP69†	NA	Copy number increase	16.70	7.50	0.53
LuCaP105	Rib	Copy number increase	119.16	70.93	0.61

† Xenograft discontinued.

* Xenograft derived from patient #9 in Table 2.

** Xenograft derived from a patient with localized prostate cancer.

Only samples successfully hybridized with at least one marker were presented in the table, including 23 with *TMPRSS2*/*ERG* FISH, 26 with *AR* FISH, and 25 xenografts with *PTEN* FISH results.

### Intra- and Inter-patient Comparison of Genomic Aberrations and Heterogeneity in Castration Resistant Prostate Cancer

**Figure 3:**
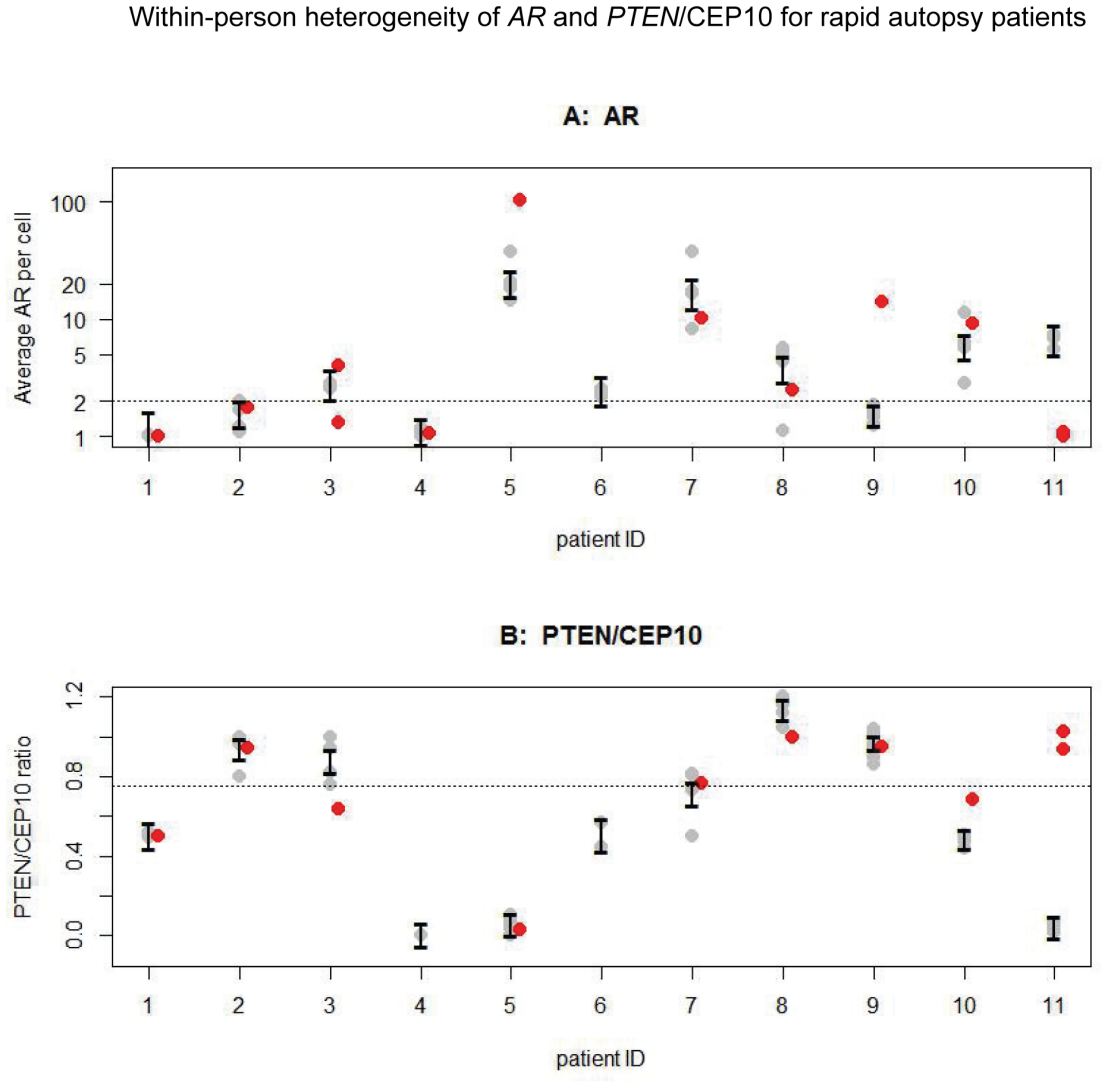
Within-patient heterogeneity of *AR* and *PTEN*/CEP10 for rapid autopsy patients (n = 11). Each tumor’s FISH result is represented by a plotting character (grey for metastatic lesions, red for prostate) with multiple lesions in the same patient at the same X coordinate. Confidence intervals for subject-specific average copy number values are shown in black. Thresholds for abnormal signals are marked as horizontal dashed lines on each plot. (A) Average number of *AR* per nucleus. (B) Average *PTEN*/*CEP10* ratio.

The 3-marker FISH analyses yielded two observations from patients with metastatic prostate cancer: (1) within the same patient, aberrations in metastatic tumors were generally consistent across tumors; (2) several primary prostate tumors of CRPC patients exhibited a profile distinct from distant metastatic sites. FISH analyses for *AR* copy number (Figure 3A) and *PTEN*/CEP10 ratio (Figure 3B) showed discordant results between the primary tumors and metastatic lesions. In particular, for patient #9, the prostate tumor showed *AR* copy number increase, whereas the metastatic lesions all had average *AR* <2. For patient #11, the primary prostate tumors showed normal *AR* results while metastatic lesions showed *AR* gain. Similarly, the prostate lesion in patients #3 demonstrated heterozygous *PTEN* deletion when all metastatic lesions had normal *PTEN*. In contrast, for patient #11, the metastatic lesions showed homozygous *PTEN* deletion while the prostate lesion did not. In other cases (#8 and #10), prostate tumors did not differ from metastatic lesions in abnormal *vs* normal marker signals, but were outside of the 95% confidence interval for the subject-specific average based on linear mixed models fit to metastatic lesions.

In general, more than 75% of the variability was between-patient, with relatively little within-patient variation: the intraclass correlation coefficient was 0.76 (95% CI 0.54–0.90) for average *AR* and 0.82 (95% CI 0.62–0.92) for *PTEN*/CEP10. However, when the entire panel was evaluated for each individual, 4 (#3, #5, #9 and #11) out of 8 patients (50%) with data available from all three markers in the local prostate tumors showed different profiles between the primary and metastatic tumors. Further comparison using data of individual markers showed different levels of deviation of the primary from metastatic tumors. Of the 8 patients with available *TMPRSS2*/*ERG* FISH results on prostate site tumors, 3 (37.5%) had results in the prostate different from those in other metastatic sites (Table 2). Interestingly, patient #5 demonstrated dual deletion fusion among metastatic sites, while only single deletion fusion was detected in a tumor from the prostate of the same patient. In the analyses of *AR*, 3 (#3, #9, and #11) out of 10 patients (30%) showed results in the prostate that deviated from extra-prostatic tumors. The assessment of *PTEN* deletion showed that 2 (#3 and #11) out of 9 patients (22.2%) demonstrated different *PTEN* FISH results between prostate site and metastatic tumors. In patient #11, all extra-prostatic metastasis showed homozygous *PTEN* deletion, while prostate tumors showed normal *PTEN* results. Similarly, the panel data for xenografts also indicated that xenograft lines derived from the same patient tend to show the same genomic abnormality (Table 3).

### Intra-tumoral Assessments of Genomic Heterogeneity in Metastatic Patients

We next sought to evaluate variation in genomic alterations detected by the FISH panel in individual cells comprising a primary or metastatic tumor. We found substantial intratumor variation in *AR* copy number for prostate site tumors. Linear mixed models predicted *AR* copy number at the cell level by tumor type, with random patient effects. Table 4 shows estimates for the number of *AR* per cell, and for the covariance parameter estimates that show how within-tumor variation and measurement error differ between tumor types. Prostate site tumors had the highest estimated within-person *AR* standard deviation (1.43 *AR* per cell). Several prostate tumors and lymph node metastasis had some unusually high counts that may have contributed to the estimate. For the *PTEN/CEP10* ratio, the covariance estimates were also found to be heterogeneous by tumor type (χ^2^_6_ = 20, p = 0.003), but within-patient prostate *PTEN/*CEP10 intratumoral heterogeneity was not different from that of metastasis (χ^2^_1_ = 0.7, p = 0.40). Table 4 suggests that the tissue-based heterogeneity differences were due to low within-patient variation in the peritoneal and adrenal lesions. These effects may be confounded with patient effects, since few patients had adrenal or peritoneal lesions. By a likelihood ratio test, statistical models with separate covariate estimates for each tumor type fit the data better than a model that did not distinguish between tumors (χ^2^_6_ = 412, *P*<0.001), and a model that distinguished between prostate tissue and other lesions (χ^2^_5_ = 332, *P*<0.001).

**Table 4:** Summary of intratumoral heterogeneity.

		*AR* (N = 2591 cells in 11 patients) patients			*PTEN*/*CEP10* (N = 722 cells in 11 patients)		
	N^1^	Average *AR*per cell^2^	Predicted AR percell (95% confidenceinterval)	Predicted within-patient standarddeviation	Average*PTEN*/*CEP10* 1	Predicted *PTEN*/*CEP10* (95%confidence interval)	Predicted within-patient standarddeviation
Prostate	12	2.1	3.0 (2.6–3.3)	1.43	0.9	0.8 (0.7–0.9)	0.43
Adrenal	2	1.6	1.5 (1.4–1.6)	1.04	1.0	1.0 (0.9–1.1)	0.21
Liver	6	2.6	2.3 (2.1–2.5)	1.12	0.7	0.7 (0.6–0.8)	0.44
Lymph Node	34	5.3	3.5 (3.3–3.7)	1.29	0.5	0.7 (0.6–0.7)	0.47
Lung	10	2.0	2.1 (2.0–2.3)	1.13	1.0	0.7 (0.6–0.8)	0.46
Peritoneal	1	2.2	2.0 (1.7–2.3)	1.06	0.4	0.4 (0.2–0.6)	0.29
Spleen	2	1.3	1.3 (1.2–1.3)	1.02	0.4	0.5 (0.3–0.7)	0.54

1 Number of tumors in sample.

2 Median for analyzed tissue.

Predicted values and covariance parameter estimates are from linear mixed models predicting copy number by tumor type, with random patient effects and separate covariance parameter estimates (within-patient heterogeneity and measurement error) for each tumor type.

### Correlation of Genomic Alterations and Gene Expression in Castration Resistant Prostate Cancer

In order to investigate the functional relationship of genetic aberrations detected by our panel, we correlated our FISH findings with gene expression data from 91 matching Met/CRPC samples, including 65 patient tumors and 26 xenografts (Table S1).

**Figure 4:**
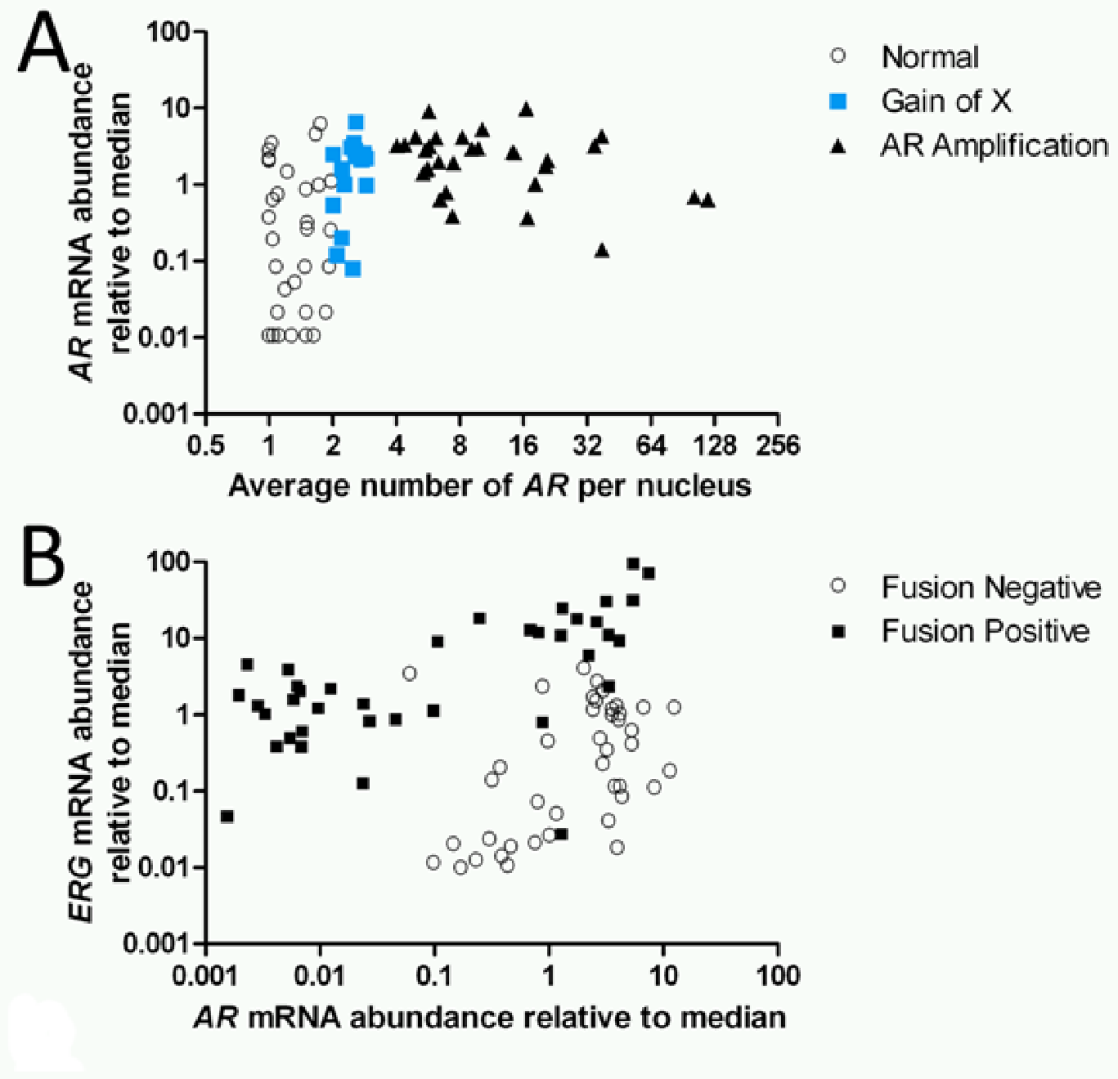
The correlation between changes in gene expression and aberrations detected by the panel. (A) Scatter plot demonstrates the correlation between FISH and expression data of *AR* (n = 88). The X-axis denotes the average number of *AR* signals per nuclei. The Y-axis denotes the *AR* mRNA level detected in the expression array relative to the median. Black open circles denote samples with an average of less than 2 *AR* per nuclei. Blue squares denote samples with copy number gain of *AR* due to gain of X. Black triangles denote samples with *AR* gene amplification. (B) Scatter plot demonstrates the effect of *TMRPSS2*:*ERG* fusion and *AR* expression on the abundance of *ERG* transcript (n = 80). The X- and Y-axis denotes relative mRNA abundance of *AR* and *ERG* compared to the median, respectively. Open circles and filled squares represent tumors without and with *TMPRSS2*:*ERG* fusion, respectively.

We first compared *AR* copy number, determined by FISH, with the *AR* transcript abundance, determined by cDNA microarray, from the same tumor sample. We observed a wide range of *AR* expression in Met/CRPC tumors (Figure 4A). The average number of *AR* per nucleus and the level of relative *AR* mRNA were positively correlated with rho = 0.52 (*P*<0.001) (Figure 4A). When normalized to the median *AR* mRNA expression level of all tumors with both *AR* FISH and mRNA expression data (n = 88), samples with *AR* gain (n = 48), including gain of X (n = 17) and *AR* amplification (n = 31) expressed *AR* mRNA at 2.5±0.3-fold (Mean±S.E.) higher than the median, while tumors without *AR* gain (n = 40) had *AR* mRNA level as 0.7±0.2-fold comparing to the median (W = 1106, *P*<0.001). When tumors with *AR* gain were further divided into groups of gain of X (n = 17) vs *AR* gene amplification (n = 31), our data showed that *AR* mRNA was expressed at a similar level between the two (W = 361, *P = *0.24).

We then assessed the effect of *TMPRSS2*:*ERG* fusion on *ERG* mRNA levels and evaluated whether *ERG* expression also associated with the *AR* abundance in Met/CRPC tumors (n = 80, Figure 4B). Fusion-negative tumors (n = 42) expressed *ERG* mRNA at 0.7±0.1 relative to the probe median, while fusion-positive tumors (n = 38) expressed significantly higher *ERG* mRNA at 910.8±3.2 fold relative to probe median (W = 2073, *P*<0.001). Copy number increase (CNI) of *ERG* (or of both *TMPRSS2* and *ERG* without fusion) did not associate with higher *ERG* mRNA expression.

As *ERG* expression in the context of a *TMPRSS2:ERG* fusion is regulated by AR activity, we evaluated the effect of *AR* on *ERG* expression in Met/CRPC tumors with and without *TMPRSS2*:*ERG* fusion. While both the fusion-positive and fusion-negative samples showed a significant correlation between *AR* mRNA and *ERG* mRNA expression, this correlation appeared stronger in fusion-positive samples (rho = 0.64, *P*<0.001, n = 38) than in fusion-negative samples (rho = 0.36, *P = *0.02, n = 42). This correlation was further confirmed by a dichotomized comparison of *ERG* expression levels for the 38 fusion-positive samples between low- and high- *AR* mRNA expression groups using the median probe intensity as a divider. The low *AR* expressing tumors (n = 20) expressed *ERG* at 3.6±1.1 fold relative to the probe median, while the high *AR* expressing tumors (n = 18) expressed *ERG* at 21.2±5.8 fold of probe median (*P*<0.01).

## Discussion

### A Three Marker FISH Panel Detects High Rates of Recurrent Genomic Aberrations in Localized and Metastatic Prostate Cancers

Because of the controversial prognostic utility of *TMPRSS2*:*ERG* fusion in prostate cancer, we employed the strategy of a three-marker FISH panel to detect well documented prostate cancer DNA aberrations, including *TMPRSS2*/*ERG* rearrangements, *AR* copy number gain, and *PTEN* deletion. This panel clearly detected a significant number of genetic abnormalities in prostate carcinomas, 53% in primary tumors and 87% in Met/CRPCs. At the individual tumor level, the odds of a Met/CRPC tumor being abnormal were 4.5 times greater than that for a primary tumor. Collectively, if aberrations in these genomic loci associate with aggressive tumor behavior, then this three-marker FISH panel may be a useful tool in distinguishing high-risk patients from low-risk ones at diagnosis or in repeat assessments using active surveillance strategies. In addition, this approach may be particularly useful in characterization of circulating and disseminated tumor cells (CTC/DTC) as using fewer cells for analysis and getting data on three specific markers would be a significant advantage. The utility of these three markers is further supported by findings from a recent study using whole exome and transcriptome sequencing technologies [21]. Grasso et al. identified that *AR* and *PTEN* had the highest level of copy number gains and losses, respectively, in prostate cancer, especially CRPC. Their integrated genomic approach also demonstrated the interplay of these genomic alterations with *TMPRSS2/ERG* rearrangements. For each individual marker, our study detected similar abnormality rates as reported in the literature. For rearrangements of *TMPRSS2* and/or *ERG*, previous findings showed *ERG* rearrangements in 30–50% of localized prostate cancers [1], [2], [4], [5], [22] and 40–50% of metastatic diseases [4], [23]–[25]. With our novel 4-color FISH technique, capable of detecting rearrangements of *TMPRSS2* and/or *ERG* simultaneously in a single hybridization, we found the similar prevalence for *TMPRSS2*:*ERG* fusion, as well as non-fusion alternative rearrangements in 10–12% patients in both groups. However, dual/complex *TMPRSS2*:*ERG* fusion, which has been shown to associate with poor survival, occurs with a substantially greater frequency in Met/CRPC patients (17%) than in primary cancer patients (3%). Similarly, copy number increase (CNI) of *TMPRSS2* and *ERG* without fusion was more frequent in Met/CRPC patients (33%) than in primary cancer patients (3%), suggesting increased genetic instability as the disease progresses, which was also observed in our studies of disseminated tumor cells obtained from prostate cancer patients [23], [26].

To date, multiple studies have demonstrated the occurrence of *PTEN* loss ranging from less than 20% to nearly 70% in early stage prostate cancer [8], [11], [27], [28]. The variation could be attributed to multiple factors such as differences in patient populations, cohort sizes, and the cutoffs used to determine the *PTEN* deletion. Setting the cutoffs (based on the percentage of abnormal nuclei among all nuclei scored) as 10% for homozygous and 40% for heterozygous deletion, Reid and colleagues identified 17% of untreated primary prostate cancers exhibiting heterozygous or homozygous deletion of *PTEN* [11]. Setting the cutoffs as 30% for homozygous and 20% for heterozygous deletion, Yoshimoto et al. identified the presence of heterozygous and homozygous *PTEN* deletion in 39% and 5% prostate cancer patients, respectively [8]. We observed *PTEN* deletion in 21% of primary cancer patients and 47% of the CRPC patients based on the average ratio of *PTEN*/CEP 10 signals. Similar to previous findings [29], our study found that *PTEN* deletion tumors also tended to harbor *TMPRSS2/ERG* abnormalities (Figure 2E).

Our findings on *AR* gene amplification are unique and particularly interesting. *AR* amplification is generally considered to be only associated with CRPC tumors, induced by hormonal deprivation therapy or treatment with AR antagonists. Previous FISH studies rarely detected *AR* gene amplification in clinically localized prostate tumors before hormonal therapy, but gain of the X-chromosome has been reported in 30–50% patients when a cutoff for gain was set at 9.8% of all cells examined [30], [31], which implied that an average of > = 1.1 copies of the X-chromosome per nucleus were considered abnormal. In recurrent prostate cancer, *AR* amplification was common, with the reported frequency varying between 20% and 60% [6], [7], [32], [33]. In these studies, *AR* gene amplification was defined in a slightly different manner. For example, among the studies that used *AR*/X ratio to define the amplification, the cutoffs vary from 1.5 [32], [33], 2.0 [6], to 3.0 [7]. In the present study, we separated the subtype of true *AR* gene amplification, defined as *AR*/X ratio > = 2.0, from general *AR* gain, defined as having an average *AR* per nucleus of > = 2.0. We found *AR* gain in 58% of Met/CRPC patients, including 39% presenting as true *AR* gene amplification and 19% demonstrating *AR* gain due to simultaneous gain of the X-chromosome, with an average number of X-chromosomes per nuclei exceeding 2.0 (Table 1). There was, however, no difference in the *AR* mRNA expression between the groups of X-gain vs *AR* gene amplification; *AR* copy numbers correlated well with *AR* mRNA levels (Figure 3A). We also observed by SNP-array CGH analysis that the multiple X centromere signals observed by FISH sometimes represent only focal gain or amplification of the genomic region around the centromere of the X-chromosome including *AR* rather than gain of the entire X-chromosome (Schoenborn, unpublished data not shown). These data argue for using the absolute *AR* copy number alone to define *AR* gain/amplification in FISH studies regardless of the *AR*/X ratio. Consequently, using the cutoff of 2.0 for *AR* gain would mean that a tumor would be considered abnormal for *AR* gain when *AR* copy number is at least doubled (from one copy in normal male cells to two copies in cancer cells) in 100% of the cells, which might be too stringent a criterion and explains why *AR* gain was never reported previously in primary prostate cancer. Our experimental cutoff based on signal patterns seen in a series of normal controls was 1.48. Therefore, we used the 1.50 cutoff for moderate *AR* gain in Table 1, which translates to that *AR* gain in 50% of the cells would be considered abnormal. With this cutoff, we observed 6% of primary patients and 77% of Met/CRPC patients with *AR* gain. This should be a better definition for *AR* gain and may allow identification of primary prostate cancer patients with high risk for disease progression. Supporting evidence came from LuCaP 96 (Table 3), a xenograft line derived from a localized primary prostate cancer which showed moderate *AR* gain (1.52 *AR* per nucleus). Its castration-resistant derivative line LuCaP 96CR showed clear *AR* amplification (5.72 *AR* per nucleus). The original patient indeed had aggressive disease and died from prostate cancer. The caveat, however, is that the xenograft data may not faithfully represent the original tumor genomics due to potential selection pressures over time on the xenograft specimens.

### FISH Detected Genetic Abnormalities Strongly Correlate with Changes at the Expression Level and Suggest Functional Interactions between *AR*, *PTEN* and *TMPRSS2/ERG*

Chaux *et al.* identified a strong association between ERG protein staining using immunohistochemistry and the *TMPRSS2*:*ERG* fusion status defined by FISH [34]. Similarly, our study showed that *ERG* mRNA expression was significantly correlated with the presence of *TMPRSS2*:*ERG* fusion. We also demonstrated a strong positive correlation between *AR* copy number gain and increased level of *AR* mRNA expression, supporting previous studies which showed higher levels of AR protein expression in prostate tumors with *AR* gene amplification than tumors without *AR* amplification [33]. Unlike this study that did not find an effect of X-chromosome gain on *AR* mRNA, we found higher *AR* mRNA levels in tumors with simple gain of X-chromosome, the amplitude of which could not be differentiated from tumors with true *AR* gene amplification. The similarity in *AR* mRNA levels in these two groups may in part be due to the nature of transcriptome array analyses, where the quantification of very high levels of *AR* mRNA reaches a plateau.

Related to the functional interactions of these genetic aberrations, previous studies demonstrated the cooperative relations between *PTEN* deletion and *TMPRSS2*/*ERG* rearrangements in animal models [29], [35]. Clinical studies demonstrated significant correlations between *PTEN* gene deletion and deregulation of p-AKT as well as AR protein expression in advanced localized prostate cancer [9]. Two recent studies suggested cross-talk between androgen signaling pathway and the PI3K signaling in a reciprocal fashion [12], [13]. At the genomic level, studies using large clinical cohorts demonstrated both presence and absence of enrichment between *TMPRSS2*:*ERG* fusion and *PTEN* gene deletion in prostate cancer [9], [11]. Our study also confirmed enrichment of *TMPRSS2/ERG* abnormalities in tumors with either *PTEN* deletion or *AR* gain (Figure 2E). *AR* gain, but not *PTEN* deletion, was enriched in Met/CRPC tumors with *TMPRSS2*/*ERG* abnormalities. However, it is not obvious from our study that *PTEN* and *AR* expression were inversely correlated in prostate cancer, as previously reported [14].

More importantly, we demonstrated that *AR* and *ERG* expression levels strongly correlated with each other, especially in *TMPRSS2:ERG* fusion-positive tumors (Figure 4B). We propose the model that moderate *AR* gain in a *TMPRSS2:ERG* fusion-positive primary prostate cancer might synergistically enhance the expression of *ERG*, which gives growth advantage to those cells with moderate *AR* gain. *ERG* expression beyond a certain threshold would convey castration resistance to the tumor cells, which in turn increases the *AR* copy number and expression to compensate for androgen deprivation, contributing to disease progression and metastasis. Future work is needed to further study the hypothesis and the prognostic utility of the three-marker FISH panel.

### Heterogeneity of Genetic Aberrations Detected by the Three-marker FISH Panel

The genetic heterogeneity assessment among CRPC patients showed that the major variability were between-patient. Within a given CRPC patient, aberrations in metastatic tumors were generally consistent across tumors, which are congruent with the general notion that metastatic cancer cells originated from the primary cancer cells and, therefore, likely maintain the same genetic lesion. However, some primary tumors may differ from metastatic lesions (Figure 3). This observation supported previous findings which demonstrated that primary prostate cancer is multiclonal, but most prostate cancer metastases are likely monoclonal in origin [23], [36]. Also, primary tumors in the CRPC patient population have been exposed to aggressive therapy, which over time could result in genomic alterations inconsistent with the original primary tumor. In addition, intra-tumor variation was evident by both the *AR* and *PTEN* markers, which showed greater heterogeneity from tumors at the prostate site than distant metastases. This does not negate the significant intra-patient protein expression observed in our previously reported studies [37]. These findings support the multifocal and possibly multiclonal nature of advanced stage prostate cancer, especially at the prostate microenvironment [38].

In summary, we evaluated both primary cancer patients and Met/CRPC patients for the presence of *TMPRSS2*/*ERG* rearrangements, *AR* gene copy number gain, and *PTEN* deletion using a three-marker FISH panel. Our panel detected highly recurrent genetic abnormalities that showed distinct distribution between primary prostate cancer patients and Met/CRPC patients. Since these abnormalities occurred more frequently in Met/CRPCs, which represent more aggressive disease, when present in localized primary prostate cancer, would convey aggressive characteristics to these localized tumors. Therefore, our results support the prognostic potential of the three-marker FISH panel for risk stratification. FISH findings strongly correlated with the transcriptome levels and provided further insight in the interaction of these three gene related functional pathways. Tumor heterogeneity analysis demonstrated more inter-patient variability than intra-patient, and that the intra-patient tumor heterogeneity was mainly due to the deviation of the prostate site tumor from metastases. Future studies will focus on applying this panel to retrospective or prospective studies on untreated primary cancer patients and on CTC/DTC to test its ability to stratify patients and predict clinical outcome.

## Supporting Information

Table S1 **Shows the mRNA expression results of** ***AR*** **and** ***ERG*** **of all Met/CRPC tumor samples used in this study.**
(DOCX)
