# Supplementary material for: A Three-Marker FISH Panel Detects More Genetic Aberrations of AR, PTEN and TMPRSS2/ERG in Castration-Resistant or Metastatic Prostate Cancers than in Primary Prostate Tumors
Source: PLoS One. 2013 Sep 30;8(9):e74671. doi: 10.1371/journal.pone.0074671 (PMC3787014; doi:10.1371/journal.pone.0074671)
Supplement: Table S1 — Shows the mRNA expression results of AR and ERG of all Met/CRPC tumor samples used in this study. (DOCX) [file pone.0074671.s001.docx]

Table S1: Microarray signal intensities for *AR* and *ERG* of all tumor samples

| Patient / Sample ID | Tissue | *ERG* mRNA | *AR* mRNA | *AR* gene gain | *TMPRSS2:ERG* gene fusion |
| --- | --- | --- | --- | --- | --- |
| 1 | Liver | 140 | 308392 | NA | no |
| 1 | LN1 | 49 | 273357 | no | no |
| 1 | LN2 | 139 | 341568 | no | no |
| 1 | Prostate | 1810 | 212676 | no | no |
| 2 | LN1 | 1057 | 2226 | no | yes |
| 2 | LN2 | 120807 | 445414 | no | yes |
| 2 | LN3 | 1443 | 8075 | no | yes |
| 2 | Lung1 | 23060 | 143207 | no | yes |
| 2 | Lung2 | 39029 | 259417 | yes | yes |
| 2 | Prostate | 91376 | 606340 | no | yes |
| 3 | Liver | 134 | 680269 | yes | no |
| 3 | LN1 | 420 | 264142 | yes | no |
| 3 | LN2 | 584 | 229423 | yes | no |
| 3 | Lung | 3232 | 218832 | yes | no |
| 3 | Prostate 1 | 1028 | 338141 | yes | no |
| 3 | Prostate 2 | 4181 | 5066 | no | no |
| 4 | Liver | 1115 | 3771 | no | yes |
| 4 | LN1 | 488 | 564 | no | yes |
| 4 | Lung1 | 775 | 571 | no | yes |
| 4 | Lung2 | 7746 | 1289 | no | NA |
| 4 | Spleen | 5845 | 190 | no | yes |
| 4 | Prostate | 157 | 7846 | no | NA |
| 5 | LN1 | 7654 | 181289 | yes | yes |
| 5 | LN2 | 21142 | 212648 | yes | yes |
| 5 | LN3 | 40260 | 442943 | yes | yes |
| 5 | LN4 | 35 | 105662 | yes | yes |
| 5 | LN5 | 14279 | 269098 | yes | yes |
| 5 | Prostate | 1028 | 71712 | yes | yes |
| 6 | LN1 | 2480 | 241328 | yes | no |
| 6 | LN2 | 1584 | 320019 | yes | no |
| 6 | LN3 | 1181 | 291522 | yes | no |
| 6 | Peritoneal | 4881 | 166673 | yes | no |

Supplementary table 1: Microarray signal intensities for *AR* and *ERG* of all tumor samples(continued)

| Patient / Sample ID | Tissue | *ERG* mRNA | *AR* mRNA | *AR* gene gain | *TMPRSS2:ERG* gene fusion |
| --- | --- | --- | --- | --- | --- |
| 7 | LN1 | 495 | 432180 | yes | no |
| 7 | LN2 | 1492 | 1022094 | yes | no |
| 7 | LN4 | 12 | 13965 | yes | no |
| 7 | Prostate | 1501 | 549836 | yes | no |
| 8 | Liver | 1418 | 292643 | yes | no |
| 8 | LN2 | 749 | 434217 | yes | no |
| 8 | LN3 | 221 | 930790 | yes | no |
| 8 | Lung | 1247 | 341202 | yes | no |
| 8 | Prostate | 101 | 359506 | yes | no |
| 9 | Adrenal1 | 1570 | 790 | no | yes |
| 9 | Adrenal2 | 4953 | 434 | no | yes |
| 9 | Liver | 496 | 342 | no | yes |
| 9 | LN1 | 1770 | 1974 | no | yes |
| 9 | LN2 | 2637 | 548 | no | yes |
| 9 | LN3 | 2790 | 1022 | no | yes |
| 9 | LN4 | 634 | 447 | no | yes |
| 9 | Lung1 | 3043 | 517 | NA | yes |
| 9 | Lung2 | 1299 | 271 | no | yes |
| 9 | Spleen | 1680 | 237 | no | yes |
| 9 | Prostate | 3013 | 272232 | yes | yes |
| 10 | Liver | 363 | 102572 | yes | NA |
| 10 | LN1 | 541 | 168140 | yes | NA |
| 10 | LN2 | 158 | 144084 | yes | NA |
| 10 | LN3 | 736 | 203606 | yes | NA |
| 10 | LN4 | 172 | 309174 | yes | NA |
| 10 | Lung | 873 | 418698 | yes | NA |
| 10 | Prostate | 1029 | 303257 | yes | NA |
| 11 | LN1 | 544 | 81240 | yes | no |
| 11 | LN2 | 7577 | 39767 | yes | NA |
| 11 | LN3 | 2044 | 199541 | yes | no |
| 11 | Lung | 1425 | 162695 | yes | NA |
| 11 | Prostate | 1406 | 200344 | no | no |
| 11 | Prostate | 2818 | 72777 | no | no |

Supplementary table 1: Microarray signal intensities for *AR* and *ERG* of all tumor samples(continued)

| Patient / Sample ID | Tissue | *ERG* mRNA | *AR* mRNA | *AR* gene gain | *TMPRSS2:ERG* gene fusion |
| --- | --- | --- | --- | --- | --- |
| LuCaP23.1 | LN | 11632 | 8683 | yes | yes |
| LuCaP23.1CR | LuCaP23.1 | 13969 | 103868 | yes | yes |
| LuCaP23.12 | Liver | 23084 | 20196 | yes | yes |
| LuCaP35 | LN | 15182 | 66554 | yes | yes |
| LuCaP35CR | LuCaP35 | 11847 | 334648 | yes | yes |
| LuCaP49 | Omental fat met | 60 | 127 | no | yes |
| LuCaP58 | LN | 170 | 26244 | no | no |
| LuCaP69 | NA | 23 | 38231 | yes | no |
| LuCaP70 | Liver | 25 | 12020 | yes | no |
| LuCaP73 | Prostate | 14 | 8037 | no | no |
| LuCaP77 | Femur | 17 | 31631 | NA | no |
| LuCaP78 | Peritoneal | 15 | 18852 | no | no |
| LuCaP81 | LN | 13 | 35866 | no | no |
| LuCaP86.2 | Bladder | 31516 | 106494 | no | yes |
| LuCaP92 | Peritoneal | 16351 | 56148 | yes | yes |
| LuCaP93 | Prostate | 163 | 1952 | no | yes |
| LuCaP96 | Prostate | 245 | 30841 | no | no |
| LuCaP96CR | LuCaP96 | 22 | 327716 | yes | no |
| LuCaP105 | Rib | 87 | 66007 | yes | no |
| LuCaP115 | LN | 61 | 95661 | no | no |
| LuCaP136 | Acites fluid(cells) | 25 | 62423 | no | no |
| LuCaP141 | Prostate | 272 | 243084 | yes | no |
| LuCaP145.1 | Liver | 2035 | 480 | no | yes |
| LuCaP145.2 | LN | 2329 | 160 | no | yes |
| LuCaP147 | Liver | 28 | 24762 | no | no |
| LuCaP153 | NA | 32 | 83537 | no | no |
